# PD-L1, Galectin-9 and CD8^+^ tumor-infiltrating lymphocytes are associated with survival in hepatocellular carcinoma

**DOI:** 10.1080/2162402X.2016.1273309

**Published:** 2017-01-03

**Authors:** Kostandinos Sideras, Katharina Biermann, Joanne Verheij, Bart R. Takkenberg, Shanta Mancham, Bettina E. Hansen, Hannah M. Schutz, Robert A. de Man, Dave Sprengers, Sonja I. Buschow, Maddy C. M. Verseput, Patrick P. C. Boor, Qiuwei Pan, Thomas M. van Gulik, Turkan Terkivatan, Jan N. M. Ijzermans, Ulrich H. W. Beuers, Stefan Sleijfer, Marco J. Bruno, Jaap Kwekkeboom

**Affiliations:** a Department of Gastroenterology and Hepatology, Erasmus MC-University Medical Center, Rotterdam, the Netherlands; b Department of Pathology, Erasmus MC-University Medical Center, Rotterdam, the Netherlands; c Department of Pathology, Academic Medical Center, University of Amsterdam, Amsterdam, the Netherlands; d Academic Medical Center, Tytgat Institute for Liver and Intestinal Research, University of Amsterdam, Amsterdam, the Netherlands; e Department of Experimental Surgery, Academic Medical Center, University of Amsterdam, Amsterdam, the Netherlands; f Department of Surgery, Erasmus MC-University Medical Center, Rotterdam, the Netherlands; g Department of Oncology, Erasmus MC-University Medical Center, Erasmus MC Cancer Institute, Rotterdam, the Netherlands

**Keywords:** Galectin-9, hepatocellular carcinoma, HVEM, IDO, PD-L1, tissue microarrays, tumor-infiltrating lymphocytes

## Abstract

Novel systemic treatments for hepatocellular carcinoma (HCC) are strongly needed. Immunotherapy is a promising strategy that can induce specific antitumor immune responses. Understanding the mechanisms of immune resistance by HCC is crucial for development of suitable immunotherapeutics. We used immunohistochemistry on tissue-microarrays to examine the co-expression of the immune inhibiting molecules PD-L1, Galectin-9, HVEM and IDO, as well as tumor CD8^+^ lymphocyte infiltration in HCC, in two independent cohorts of patients. We found that at least some expression in tumor cells was seen in 97% of cases for HVEM, 83% for PD-L1, 79% for Gal-9 and 66% for IDO. In the discovery cohort (n = 94), we found that lack of, or low, tumor expression of PD-L1 (*p* < 0.001), Galectin-9 (*p* < 0.001) and HVEM (*p* < 0.001), and low CD8^+^TIL count (*p* = 0.016), were associated with poor HCC-specific survival. PD-L1, Galectin-9 and CD8^+^TIL count were predictive of HCC-specific survival independent of baseline clinicopathologic characteristics and the combination of these markers was a powerful predictor of HCC-specific survival (HR 0.29; *p* <0.001). These results were confirmed in the validation cohort (n = 60). We show that low expression levels of PD-L1 and Gal-9 in combination with low CD8^+^TIL count predict extremely poor HCC-specific survival and it requires a change in two of these parameters to significantly improve prognosis. In conclusion, intra-tumoral expression of these immune inhibiting molecules was observed in the majority of HCC patients. Low expression of PD-L1 and Galectin-9 and low CD8^+^TIL count are associated with poor HCC-specific survival. Combining immune biomarkers leads to superior predictors of HCC mortality.

## Abbreviations

AFP: α fetoprotein
AMC: Amsterdam Medical Center
EMC: Erasmus MC-University Medical Center
Gal-9: galectin-9
HCC: hepatocellular carcinoma
TFL: tumor-free liver
TIL: tumor-infiltrating lymphocytes
TMA: tissue microarray

## Introduction

Hepatocellular carcinoma (HCC) is a leading cause of cancer-related death.^1^ Curative treatments such as resection, local ablation or liver transplantation are only applicable in the 20% of patients with early stage disease.^2^ For selected patients with advanced disease median survival can be modestly extended with the use of sorafenib.^3^ However, cure at this stage is no longer possible.

Immunotherapeutic strategies, such as tumor vaccination, adoptive cell therapy and immune modulating antibodies, may provide alternative therapeutic options in HCC.^4,5^ Indeed, immune modulating inhibitors against CTLA-4 (Ipilimumab) or PD-1 (Nivolumab, Pembrolizumab) and PD-L1 (Atezolizumab) have already been approved for various cancers such as melanoma, lung, kidney and bladder cancer.^6-10^ These so-called immune checkpoint inhibitors interrupt immune resistance mechanisms exploited by tumors to evade natural antitumor immunity. Reported immune resistance mechanisms include, among others, the expression of molecules that suppress intra-tumoral T-cell responses by ligating inhibitory receptors on T cells, such as PD-L1, galactin-9 and HVEM and the expression of enzymes that generate T-cell inhibitory metabolites, such as IDO.^11^

Binding of PD-L1 to its receptor PD-1, on activated T cells, suppresses T-cell responses.^12^ PD-L1 is expressed in numerous tumors, including HCC, and *in-vitro* abrogation of the PD-1/PD-L1 interaction has been shown to reinvigorate tumor-specific responses of T-cells isolated from HCC patients.^13-19^ Galectin-9 (Gal-9) is a carbohydrate-binding protein that is involved in T-cell homeostasis.^20^ Contrasting effects of Gal-9 on antitumor immunity have been described. On the one hand binding of Gal-9 to its receptor TIM-3, expressed on activated T cells, causes T-cell dysfunction and apoptosis in tumors,^21^ while binding of Gal-9 to CD44 promotes the differentiation of T-regulatory cells.^22^ Conversely, Gal-9 can enhance T-helper 1 type antitumor immunity,^23^ inhibit NK cell chemotaxis to the tumor microenvironment^24^ and exert anti-metastatic potential on tumor cells.^25,26^ Like PD-L1, Gal-9 is expressed in several cancers, including HCC.^21,27^ HVEM is a “molecular switch” with dual immune-stimulatory and inhibitory functions.^28^ By ligation of LIGHT, HVEM stimulates T-cell responses, while binding to BTLA or CD160 leads to inhibition of T-cells. HVEM is known to be expressed in melanoma^29^ and most recently in HCC.^30^ IDO is the rate-limiting enzyme in the catabolism of the essential amino-acid tryptophan.^31^ Tryptophan depletion, as well as accumulation of tryptophan catabolites such as kynurenine, induce T-cell anergy and apoptosis. IDO inhibitors are currently in clinical development. IDO expression has been demonstrated in several cancer types, including HCC.^32^

As clinical efficacy of immune checkpoint antibodies seems dependent on expression of their target molecules in tumors^33,34^ knowledge of their expression patterns in HCC may establish which of these molecules, or combinations, might be promising to target. No published study has systematically examined co-expression of multiple immune inhibitory molecules in a homogeneous cohort of HCC patients before. Thus, the primary aim of the present study was to examine the patterns of co-expression, as well as the relationship with the tumor-infiltrating lymphocytes (TIL) and cancer-related survival, of several immune inhibitory molecules in HCC tumor tissue and adjacent non-tumorous tissue. PD-L1, Gal-9, HVEM and IDO were chosen for study because their mechanism of interaction with the immune system is generally understood while at the same time a reliable primary antibody is available. Tissue microarrays were constructed, and expression of the above molecules on tumor cells and hepatocytes was examined, by immunohistochemistry, in two separate patient cohorts.

## Patients and methods

### Patient population and tissue samples

Archived formalin fixed paraffin-embedded tissue samples from 154 patients who underwent hepatic resection for HCC at Erasmus MC-University Medical Center (EMC, n = 94) or Amsterdam Medical Center (AMC, n = 60), between June 2001 and July 2014, were used for this study. Fresh frozen tissue, which was available from 20 additional patients, was used for RNA extraction. Patients with HCC were selected for the study if they had undergone surgery with curative intent. Clinical information was collected retrospectively from the electronic record. The clinical information collected included etiologic factors, HCC recurrence, patient death, cause of death, as well as known prognostic clinicopathologic characteristics, such as tumor differentiation, vascular invasion, number of lesions, largest tumor size and pre-surgical α-fetoprotein (AFP) level. The study was approved by the local medical ethical committee. In addition, the study protocol conforms to the ethical guidelines of the 1975 Declaration of Helsinki.

### Tissue microarray (TMA) construction

Three or four 0.6 mm cores were taken from the tumorous area of 154 patients and two 0.6 mm cores were taken from the corresponding tumor-free liver (TFL) area of 133 of these patients. Areas with vital tumor and TFL tissue were marked by experienced pathologists (KB or JV) using archived H&E glass slides. The TMAs were made using either an automated tissue-arrayer ATA-27 (Beecher Instruments, Silver Springs MD, USA) or a fully manual tissue-arrayer MTA-1 (Beecher Instruments).

### Immunohistochemistry and scoring

Complete information on the immunohistochemistry protocols can be found in the supplementary methods section. Complete information on the primary antibodies used can be found in Table S1. Scoring was performed by two independent investigators (KS and HS) blinded to clinical outcome and differences resolved by mutual agreement. Scoring was performed on cancer cells (tumor cores) or hepatocytes (TFL cores). Intensity was scored in a scale from zero to three. Intra-core heterogeneity of staining intensity of tumor cells or hepatocytes was rarely observed in our cohort, thus only staining intensity was taken into consideration. In the case of CD8^+^ staining, the positive cells per core were counted manually and average counts were used for analysis.

### PCR amplification

All primers for the target genes were intron spanning and sequences are listed in Table S2. The RT-PCR amplification technique and primer sources are described in detail in the supplementary methods section.

### Statistical analysis

All analysis was performed in duplicate. The discovery phase was performed in the EMC cohort while the validation phase was performed in the AMC cohort. The differences in expression of immune inhibitory molecules between tumor and TFL tissue was analyzed with the paired *T*-test. The associations between clinicopathologic parameters with the expression of immune inhibitory molecules, as well as the co-expression of the immune inhibiting molecules with each other, were examined using the χ2 tests or the *T*-test as appropriate. Survival (time to recurrence or HCC-specific survival) was calculated from the date of surgery to the date of event (recurrence or death from HCC, respectively), or the date of last follow-up. Patients lost to follow-up were censored as of the last day of follow-up. Patients who died from causes other than HCC were censored at the date of death. Survival curves were estimated by the Kaplan-Meier method. For sensitivity analysis, the survival analysis was repeated by excluding the patients who died from post-surgical complications within 3 mo after surgery. Optimal high vs. low values were established by examining a grid of cutoffs and choosing the cutoff with the lowest −2 log likelihood, taking into consideration to maximize the proportion of patients identified by the cutoff value when possible. The Breslow test was used to asses differences between survival curves of different groups, while for parameters with three or four linearly associated levels the linear trend for factor levels was used. Given that we examined the expression of five individual parameters (PD-L1, Gal-9, HVEM, IDO, CD8^+^TIL count) Bonferroni's correction required a *p*-value of < 0.01 for statistical significance in the discovery cohort. For multivariate analysis, the Cox proportional Hazard regression analysis was used. The statistical analysis was performed using the SPSS© 21 software.

## Results

### Patient cohorts and baseline clinicopathologic characteristics

Clinicopathologic characteristics of patients in the EMC discovery cohort and the AMC validation cohort can be seen in Table 1, while complete information on etiology of liver disease can be found in Table S3. Median time to recurrence and overall survival were 19.4 mo and 37.2 mo for the EMC cohort and 23.5 mo and 31.8 mo for the AMC cohort. Recurrence was seen in 78 patients while 42 patients died from HCC. A complete list with the causes of death can be found in Table S4. From the known clinicopathologic prognostic factors an AFP level above 400 µgL^−1^ (*p* = 0.002), multiple lesions (*p* = 0.043) and tumor size >3 cm (*p* = 0.024) were associated with poor HCC-specific survival in the combined cohort (Fig. S1). Tumor grade and vascular invasion were not associated with HCC-specific survival.

**Table 1.** Patient characteristics.

Characteristics	Discovery cohort N = 94	Validation cohort N = 60
Male/Female (%)	63/31 (67/33)	48/12 (80/20)
Hepatitis-B^a^/Hepatitis-C^b^ (%)	23/11 (25/12)	14/19 (23/32^*^)
Cirrhosis (%)	32 (34)	20 (33)
Tumor differentiation (1–3)	26/47/20 (28/50/22)	15/34/10 (25/58/17)
Vascular invasion	58 (68^*^)	13 (42)
Single lesions (%)	72 (77)	50 (83)
Median size (Range)	5.9 cm (0.5–25.0)	5.0 cm (1.0–29.0)
Median AFP (Range)	8.5 ug/L (1–63.000)	9.0 ug/L (2–29.000)
Recurrence	50 (53)	28 (47)
Death	44 (47)	21 (35)
HCC-related death	29 (31)	13 (22)

a HBsAg(+) and/or anti-HBc positive;

b anti-HCV positive;

* There is statistically significant more Hepatitis-C in the AMC cohort and vascular invasion in the EMC cohort.

### Description of immune inhibitory molecule expression in the combined cohorts

**Figure 1.**
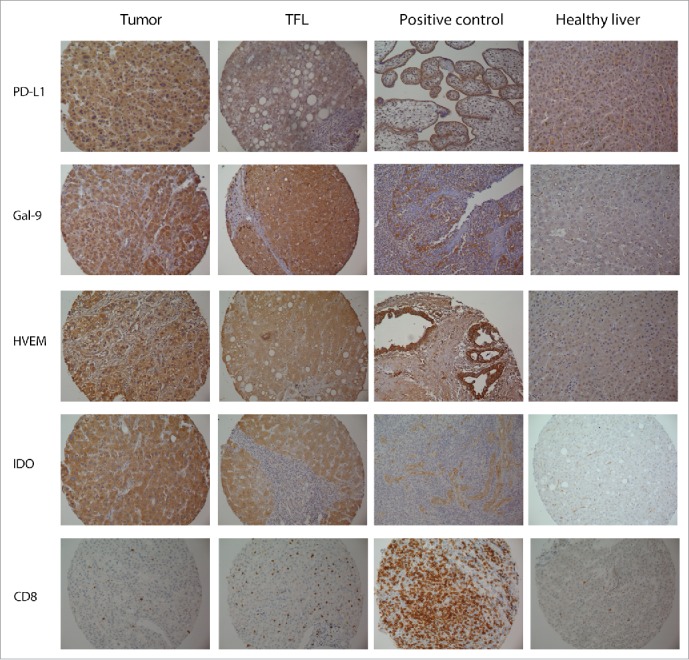
Representative stainings of tumor tissues and corresponding TFL tissues, positive control tissues and normal liver. Positive controls tissues shown are placenta for PDL1, pancreatic cancer for HVEM and tonsil for Gal-9, IDO and CD8. The positive control tissues stain as expected from prior literature. Note the hepatocyte staining seen for all molecules (except CD8^+^) in the TFL tissue area and the general lack of hepatocyte staining in normal liver tissue. For Gal-9 characteristic Kupffer cell staining can be seen in the normal liver tissue.

**Figure 2.**
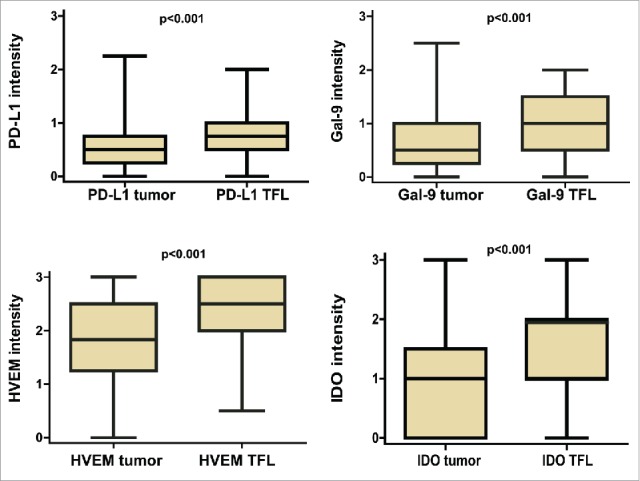
Comparison of protein expression levels of PD-L1, Gal-9, HVEM and IDO in the tumor tissues and the corresponding TFL tissues in the combined cohort. Boxplots representing protein expression in tumor cells vs. TFL tissue. *p* values were determined by the paired T-test.

Fig. 1 shows representative stainings in tumor tissue, TFL tissue, positive control tissues and healthy liver tissues, while Fig. S2 shows examples of various expression levels observed in tumor tissues, for all the molecules examined in our study. At least some cytoplasmatic expression in tumor cells was seen in 82.9% of cases for PD-L1, 78.8% for Gal-9, 96.6% for HVEM and 66.4% for IDO. In addition to their expression in the tumor tissues, PD-L1, Gal-9, HVEM and IDO were also expressed by hepatocytes in the surrounding TFL tissue. At least some cytoplasmatic expression in hepatocytes, in TFL tissue, was seen in 95.8% of cases for PD-L1, 93.5% for Gal-9, 100% for HVEM and 92.6% for IDO. No relationship was found between the expression of any molecule in tumor, or in the TFL tissue, with etiologic factors, or other clinicopathologic characteristics, after Bonferroni correction (Table S5). Tumor expression correlated with TFL tissue expression in all cases. Fig. 2 depicts the level of expression of each molecule in tumor cells and in hepatocytes in the corresponding TFL tissues for the combined cohort. Note that there is a statistically significant under-expression of all molecules in the tumor tissue. To confirm that these molecules are indeed expressed in TFL tissues, we quantified mRNA expression in paired tumor and TFL tissues of 20 resected HCC patients, from which fresh frozen tissue was available. In Fig. S3, we show that mRNA encoding for all these immune molecules is indeed expressed in the TFL tissues, at levels comparable to the levels seen in the tumor tissues. Our results indicate that the investigated immune inhibitory molecules are frequently expressed not only in the tumor but also in the TFL compartment of HCC patients.

### Immune inhibitory molecule expression: Association with survival and recurrence in the discovery (EMC) cohort

**Figure 3.**
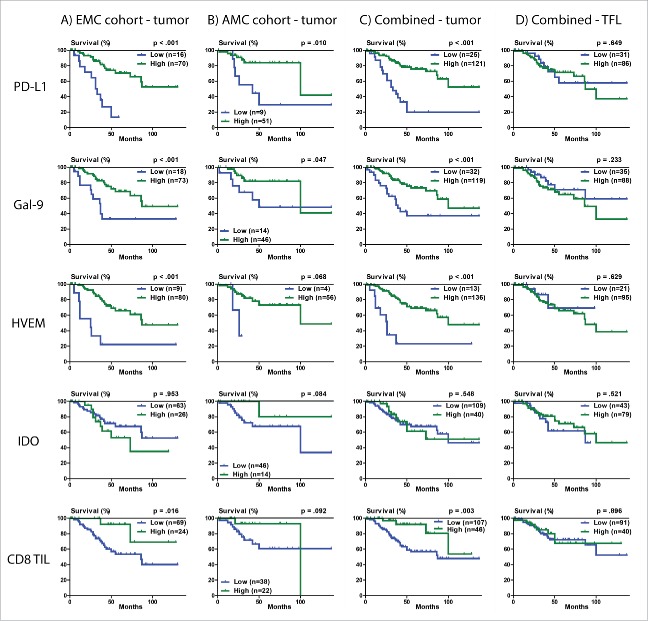
HCC-specific survival Kaplan-Meier curves of PD-L1, Gal-9, HVEM, IDO and CD8^+^TIL count in the tumor and TFL compartment. (A) Survival curves in relation to tumor expression for the discovery (EMC) cohort. (B) Survival curves in relation to tumor expression for the validation (AMC) cohort. (C) Survival curves in relation to tumor expression for the combined cohorts. (D) Survival curves in relation to TFL tissue expression for the combined cohorts. Optimal high vs. low values were established by examining a grid of cutoffs and choosing the cutoff with the lowest −2 log likelihood. For determination of the *p* values the Breslow test was used.

Next, we investigated the relationship between immune inhibitory molecule expression and survival in the EMC cohort. Optimal cut-offs for low versus high staining were made as described in the statistical methods. Patients were considered to have low PD-L1 (n = 16) or Gal-9 (n = 18) staining when there was complete absence of staining, while low HVEM included patients with either complete absence of staining or very faint staining (n = 9). Low IDO (n = 63) included patients with complete absence, or at most +1, staining intensity. We found that expression in tumor tissue, but not in surrounding TFL tissue, was significantly associated with HCC-specific survival for three out of the four molecules examined (Fig. 3A). Specifically, no or low tumor expression of PD-L1 (*p* < 0.001), Gal-9 (*p* < 0.001) and HVEM (*p* < 0.001) was associated with poor HCC-specific survival, while expression of IDO was not associated with HCC-specific survival (*p* = 0.953). Hazard ratios and 95% confidence intervals can be found in Table 2. In addition, we quantified the numbers of CD8^+^TIL. The relationship of CD8^+^TIL count with HCC-specific survival was not significant after Bonferroni's correction (*p* = 0.016). For HCC recurrence the relationship with expression of these molecules followed the same trends (Fig. S4). Low tumor expression of PD-L1 (*p* < 0.001), Gal-9 (*p* = 0.009), HVEM (*p* = 0.004), and also low CD8^+^TIL count (*p* = 0.007) were significantly associated with shorter time to HCC recurrence, while tumor expression of IDO was not associated with recurrence after Bonferroni's correction (*p* = 0.029). The results did not differ when analyses was performed by excluding the nine patients who died at EMC in the post-surgical period from causes other than HCC.

**Table 2.** Cox-proportional hazard regression analysis of patients' HCC-specific survival in the discovery cohort.

	Univariate			Multivariate		
Variables	HR	95% CI	*p*-value	HR	95% CI	*p*-value
AFP>100 ug/L	2.67	1.17–6.13	0.020	4.83	1.85–12.6	0.001
One vs. multiple lesions	3.53	1.63–7.67	0.001	2.37	1.01–5.56	0.048
PD-L1	0.19	0.86–0.43	<0.001	0.30	0.13–0.72	0.007
Gal-9	0.31	0.15–0.66	0.003	0.33	0.14–0.80	0.014
HVEM	0.21	0.09–0.50	<0.001	0.50	0.20–1.25	0.718
CD8^+^TIL count	0.22	0.05–0.93	0.040	0.18	0.04–0.82	0.027

Based on a multivariate analysis, where all parameters with *p* values < 0.02 were entered in a single model, we derived that tumor expression of PD-L1, Gal-9, CD8^+^TIL count, AFP level and number of lesions were independent predictors of HCC-specific survival in the EMC cohort (Table 2). HVEM was not independently associated with HCC-specific survival when examined together with the other immune parameters.

**Figure 4.**
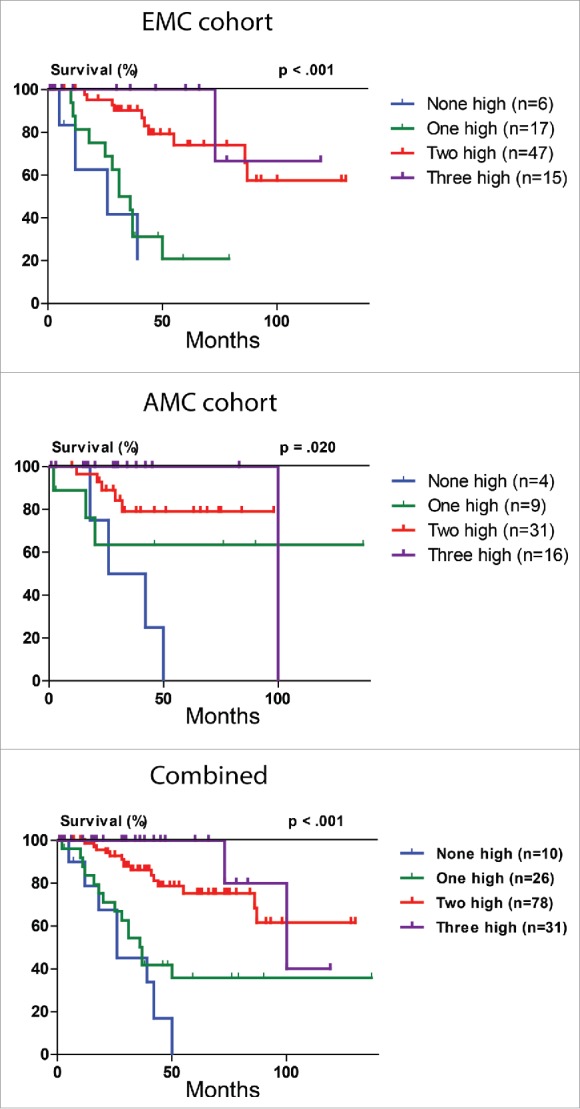
HCC-specific survival Kaplan-Meier curves of the combined PD-L1, Gal-9 and CD8^+^TIL count biomarker. Combination of PD-L1, Gal-9 and CD8^+^TIL count in relation to HCC-specific survival in the discovery (EMC) cohort, validation (AMC) cohort and combined cohort. For determination of the *p* values the linear trend for factor levels was used.

Because of the ability of the immune inhibitory molecules to predict HCC-specific survival, we wondered whether combining all three independently prognostic immune parameters would improve the prediction. Fig. 4 shows that low levels of two or three of these parameters in tumor tissue predicts poor survival, while low level of none, or only one, parameter predicts good HCC-specific survival. Concordantly, the combination of PD-L1, Gal-9 and CD8^+^TIL as a single biomarker was a powerful independent predictor of HCC-specific survival in multivariate analysis (*p* < 0.001, HR 0.29, 95%CI 0.18–0.48).

### Validation in the AMC cohort

External validation of the above findings was performed in the cohort from AMC. Low tumor expression of PD-L1 (*p* = 0.010) and Gal-9 (*p* = 0.047) were also significantly associated with poor HCC-specific survival (Fig. 3B). Low tumor CD8^+^TIL count showed a trend toward poor HCC-specific survival (*p* = 0.092), with a hazard ratio (HR 0.36, 95%CI 0.08–1.64) that was of similar magnitude and direction as in the discovery (EMC) cohort (HR 0.22, 95%CI 0.05–0.93). The combination of PD-L1, Gal-9 and CD8^+^TIL as a single biomarker (Fig. 4) predicted HCC-specific survival (*p* = 0.020, HR 0.43, 95%CI 0.24–0.77) and was validated as an independent predictor of HCC-specific survival in multivariate analysis in the AMC cohort (*p* = 0.005).

### Co-expression patterns and survival

Low levels of expression of either PD-L1, Gal-9 or HVEM were significantly associated with low levels of expression of the other two ligands (Table S6). Such a relationship was not seen with IDO. The CD8^+^TIL count showed a significant but weak correlation with PD-L1 expression (*p* = 0.046) but not with Gal-9, HVEM or IDO. Specifically, high expression of PD-L1 was correlated with a high CD8^+^TIL count and vice-versa.

**Figure 5.**
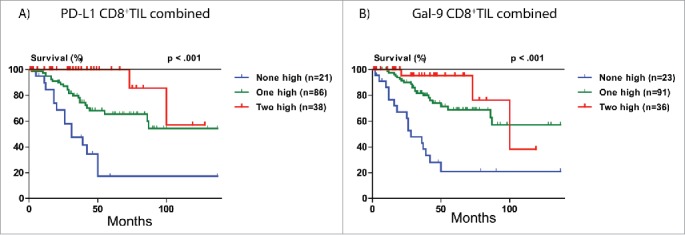
HCC-specific survival Kaplan-Meier curves of tumor PD-L1 and Gal-9 expression in relation to CD8^+^TIL count in the combined cohorts. (A) Combination of PD-L1 and CD8^+^TIL count in relation to HCC-specific survival. (B) Combination of Gal-9 and CD8^+^TIL count in relation to HCC-specific survival. For determination of the *p* values the linear trend for factor levels was used.

Having established the predictive power of the individual and combined expression of immune inhibitory molecules, we wondered how the relationship of each molecule to survival related to tumor lymphocyte infiltration. It has been previously shown that tumor expression of PD-L1 in melanoma carries different prognostic values in the setting of high vs. low TIL counts.^33,35^ Fig. 5A shows that low PD-L1 tumor expression in combination with low CD8^+^TIL count is associated with very poor HCC-specific survival, while high PD-L1 and high CD8^+^TIL count is associated with good HCC-specific survival (*p* < 0.001, HR 0.29, 95%CI 0.17–0.50). The presence of either high PD-L1 or high CD8^+^TIL count alone is associated with intermediate survival. A similar observation was made for the combination of Gal-9 tumor expression and CD8^+^TIL count (*p* < 0.001, HR 0.29, 95%CI 0.17–0.49) (Fig. 5B).

## Discussion

In two independent cohorts, we found that low tumor expression of PD-L1 and Gal-9, as well as low CD8^+^TIL count, are independent predictors of poor HCC-specific survival. HVEM expression, on the other hand, while individually an independent predictor of HCC-specific survival, was not independent when other immune parameters were taken into consideration. The combination of tumor PD-L1 and Gal-9 expression and CD8^+^TIL count appeared to be a powerful independent predictor of HCC-specific survival. We show that there is a group of patients with extremely poor prognosis who express low levels of PD-L1 and Gal-9 and have low CD8^+^TIL count. It requires a change in two of these parameters to significantly affect prognosis.

Similar to our results, low expression of PD-L1 was found to be associated with poor survival in melanoma,^33^ gastrointestinal-stromal tumors,^36^ colorectal cancer,^37^ and non-small cell lung cancer.^38^ On the other hand, other studies in melanoma,^39^ colorectal^40^ and renal cell cancer^41^ have shown the opposite. Moreover, in a third melanoma study,^42^ and in studies in squamous-cell lung cancer,^43^ urothelial cancer^44^ and breast cancer,^45^ tumor PD-L1 expression has shown no prognostic significance. In addition, and in contrast to our results, two prior studies in HCC have suggested that lower PD-L1 expression is associated with better survival,^15,18^ while one recent study reported that lower PD-L1 staining is associated with better 2-y chance of recurrence.^46^ These conflicting results on the association between PD-L1 expression and prognosis, are probably related to the lack of specificity of several anti-PD-L1 antibodies for immunohistochemical staining of PD-L1 in formalin-fixed paraffin-embedded tissues.^15,18,38,39,42^ Specifically, in the study by Gao et al.,^15^ the PD-L1 clone MIH1 was used on paraffin-embedded HCC tissue. This particular clone has been shown to be non-specific on paraffin embedded tissue in prior studies.^38,42^ To further confirm these prior observations, we stained several control tissues and TMA cores with the MIH1 clone and compared the findings to our results. We show, in Fig. S5, that the MIH1 clone failed to properly stain placenta and tonsil tissue, and also provides positive stainings of tumor cores that were found to be negative for PD-L1 using the 9A11 antibody clone. Importantly, the anti-PD-L1 antibody that we used (clone 9A11) has been extensively validated for this purpose,^41,44,47,48^ and we show that it provides the correct staining pattern in control tissues, namely selective staining of the syncytiotrophoblast layer in placenta tissue and staining of the crypt regions in tonsil tissue (Figs. 1 and Fig. S5). Moreover, in a previous study,^49^ we showed that this antibody gives similar, but more intense, staining patterns as the well-validated 5H1 clone.^50^ The use of imaging software to analyze PD-L1 density, as performed in the studies by Gao et al. and Wu et al., is another possible explanation for the differing results.^15,18^ Imaging software can inadvertently include expression of PD-L1 by stroma and TIL, in addition to tumor. Differences in the characteristics of the included patients, may be another factor. For example, the study of Calderaro et al.^46^ that examined a large cohort of patients using another validated antibody anti-PD-L1 antibody, did not report on long-term mortality endpoints, while information on recurrence was available in a little over half the patients, making direct comparisons with our study not possible. Finally, it is well known that both cytoplasmatic and membranous PD-L1 staining has been described in various cancers, and we are not the first to describe a pre-dominance of cytoplasmatic staining over membranous staining for PD-L1. In fact, studies using well-validated antibodies have demonstrated both types of staining in various cancers before^33,50^ and other prior studies in HCC have also predominately demonstrated cytoplasmatic staining.^16^ It has recently been hypothesized that cytoplasmatic tumor staining represents intracellular stores of PD-L1 ready to be transported to the membrane upon contact with immune cells.^33^ Alternatively, cytoplasmic PD-L1 may be released as a functional soluble molecule into the extracellular microenvironment.^51^

Regarding the prognostic significance of Gal-9 expression in tumors there is more consensus. Similar to our findings, under-expression of Gal-9 has been associated with poor outcome in HCC,^27^ and also in melanoma, breast, cervical and gastric cancers.^25,26,52,53^ However, one recently published study in renal cell cancer^54^ showed the opposite. It is possible that the significance of expression of Gal-9 may be tumor specific. In renal cell cancer, for example, the results for both PD-L1 and Gal-9 are opposite to ours.^41,54^

One may wonder why low expression of these molecules signifies high risk of HCC death, since their expression in itself is supposed to inhibit effector immune responses. It is known that several of these molecules are overexpressed in response to IFNγ and TIL infiltration, a process called adaptive immune resistance.^33,55-57^ Therefore, expression of immune inhibitory molecules in cancer cells may not only be induced by intrinsic mechanisms (i.e., mutations, epigenetics e.c.t.) but also by tumor-infiltrating immune cells. Probably, their presence in the tumor microenvironment reflects an active immunologic attack that is beneficial to patients. Low or no expression of such molecules could indicate that the cancer is beyond detection by the immune system or that the antitumor immune response is ineffective.

Several studies have shown that expression of PD-L1 is associated with TIL infiltration in various cancers.^33,38,43^ Our study is the first to describe this association in HCC. Stratification of tumors, based on the expression of PD-L1 and the presence or absence of TILs, has been recently proposed.^33,35^ We show that HCC patients with low tumor PD-L1 expression and CD8^+^TIL infiltration (type II tumors), which suggests tumors with immune ignorance, according to the model by Teng et al.,^35^ have the worst prognosis (Fig. 5A), whereas patients with high tumoral PD-L1 expression and TIL infiltration (type I tumors), which suggest adaptive immune resistance, have the best prognosis, as predicted. Taken together, our data demonstrate that HCC may behave according to this novel prediction model, and also suggest that type I tumors, that is tumors with an ongoing antitumor immune response (high PD-L1 and high TIL count), may be the ones to benefit from PD-L1 or PD-1 blockade. Indeed, recent observations from clinical trials show that patients who express PD-L1 are the ones that seem to benefit from anti-PD1 therapy.^34^ A similar prediction model might be true for Gal-9 in HCC, as we show in Fig. 5B. We support the idea that the use of TILs as a biomarker should be studied in the context of the expression of immune inhibitory molecules and that PD-L1 and Gal-9 are two of the molecules to be taken into consideration.

While the tumor expression status of these molecules in HCC has been reported, the expression of these molecules in the surrounding TFL tissue is much less understood. While PD-L1 and IDO have been shown, in single studies, to be indeed expressed in TFL tissue of patients with HCC,^13,58^ that has never been shown for Gal-9 or HVEM before. We here show, both by immunohistochemistry and qPCR, that all four immune inhibiting molecules are present in both the tumor and the TFL compartments. We did not observe under-expression of mRNA levels in tumor tissue compared with TFL, while such an under-expression was seen by IHC. Possible explanations for the difference could be that we examined mRNA expression only in 20 paired samples. Another explanation could be translational and post-translational regulation of mRNA. The presence of these molecules in the TFL tissue of HCC patients may be explained by the production of cytokines by the infiltrating lymphocytes.^33,55-57^ This observation suggests that therapies targeting these molecules may not only enhance antitumor immune responses but also anti-hepatitis-B virus or anti-hepatitis-C virus immune responses and thereby contribute to viral clearance. Conversely, such treatments harbor the theoretical danger of evoking undesired immunologic effects.

In our opinion, our study has several strong characteristics. We have studied a homogenous group of patients, have studied the expression of multiple immune inhibitory molecules in tumor, as well as surrounding TFL, tissue and have validated our findings in an independent cohort. Our study is the first to examine the co-expression of multiple immune inhibitory molecules in HCC. There are of course also limitations in our study. Our selected panel of immune inhibitory molecules is by no means exhaustive. We focused on molecules for which there is solid evidence for their immune inhibitory role in cancer, for which a reliable antibody was available for immunohistochemistry and for which the corresponding mechanism of interaction with immune cells is well understood. However, as our understanding of the immune inhibiting mechanisms of cancer expands other molecules should be added. In addition, while the use of TMAs to study such questions has clear benefits, such as the rapid analysis of large number of tissue cores over identical experimental conditions and preservation of valuable patient tissue for future studies, there are also drawbacks. One is the inability of TMAs to represent the complex spatial interactions of immune inhibiting molecules in the complete tumor microenvironment. For this reason, we did not evaluate the expression of immune inhibitory molecules by TIL, as performed, for PD-L1, in the study by Calderaro et al.,^46^ but focused on the expression of these molecules in tumor cells. Depending on the types of immune cells under study the relationship of expression of these molecules to outcome may be affected.^59^ Another limitation of TMAs is that it is not ideal to evaluate the presence of uncommon cell types. While CD8^+^ lymphocytes are abundant, FoxP3^+^ T-regulatory cells are much less common and thus more difficult to systematically evaluate using TMAs.

In conclusion, we show that low tumor expression of PD-L1 and Galectin-9, as well as low CD8^+^TIL count, are associated with poor HCC-specific survival in patients with resected HCC. PD-L1 and Galectin-9 expression in tumors may be induced in response to immunologic pressure which may explain why their presence is associated with prolonged survival. PD-L1 and Galectin-9 may be promising immunotherapeutic targets in HCC patients with tumors expressing these co-inhibitory molecules.

## Supplementary Material

KONI_A_1273309_s02.pdf
