# Supplementary material for: PD-L1, Galectin-9 and CD8+ tumor-infiltrating lymphocytes are associated with survival in hepatocellular carcinoma
Source: Oncoimmunology. 2017 Jan 3;6(2):e1273309. doi: 10.1080/2162402X.2016.1273309 (PMC5353918; doi:10.1080/2162402X.2016.1273309)
Supplement: KONI_A_1273309_s02.pdf [file koni-06-02-1273309-s001.pdf]

Supplementary information

PD-L1, Galectin-9 and CD8$^+$ tumor infiltrating lymphocytes are associated with survival in Hepatocellular Carcinoma

Kostandinos Sideras, Katharina Biermann, Joanne Verheij, Bart R. Takkenberg, Shanta Mancham, Bettina E. Hansen, Hannah M. Schutz, Robert A. de Man, Dave Sprengers, Sonja I. Buschow, Maddy C.M. Verseput, Patrick P.C. Boor, Qiuwei Pan, Thomas M. van Gulik, Turkan Terkivatan, Jan N.M. Ijzermans, Ulrich H.W.Beuers, Stefan Sleijfer, Marco J. Bruno, Jaap Kwekkeboom*

*Corresponding author

Department of Gastroenterology and Hepatology, Erasmus MC - University Medical Center, Room Na-1009, 3015 CE Rotterdam, The Netherlands Tel: +31-10-7034776; Fax: 31-10-7032793; Email: j.kwekkeboom@erasmusmc.nl

Table of contents

2-3 Supplementary Methods
4-8 Supplementary Tables
9-13 Supplementary Figures

Supplementary methods

Immunohistochemistry: 4µm thick sections mounted on Superfrost PlusTM slides. The sections were deparaffinized and rehydrated. Endogenous peroxidase activity was blocked with 0.3% H_2O_2 for 15 minutes. Antigen retrieval was performed in a microwave for 10 minutes using the appropriate antigen retrieval buffer for each antigen. After serum block, primary antibodies were applied at 4°C overnight (PD-L1 clone 405.9A11, kindly provided by Dr. Gordon J. Freeman, HVEM clone 2G6-2C7, IDO clone 10.1, Gal-9 goat polyclonal). Complete information on the primary antibodies and antigen retrieval used can be found in Supplementary Table 1. HRP-conjugated anti-mouse or anti-goat IgG polymer secondary antibody (EnvisionTM, DAKO) was then applied for 1 hour, followed by diaminobenzadine (DAB) as the chromogen detection method. The slides were stained with haematoxylin followed by dehydration. The above protocol was used for all antibodies with the exception of CD-8 (clone C8/144b) where an automated BenchMark ULTRA™ instrument was used in a clinical laboratory setting. Negative controls consisted of omission of the primary antibody and appropriate positive control tissues were used to evaluate specificity of all antibodies.

Quantitative RT-PCR: Fresh liver tissue was stored in Trizol (Qiagen, Valencia, USA) at -80°C until RNA isolation. Total RNA was extracted by mechanical disruption of the tissue in Trizol. After chloroform extraction, RNA was precipitated in propanol, washed with 75% ethanol and dissolved in RNase-free water. cDNA was prepared using PrimeScriptTM RT Master Mix (Takara Bio Inc.) according to the manufacturer's protocol. The SYBR Green based real time reverse transcriptase-PCR was performed using a StepOnePlus Real-Time PCR system (Applied Biosystems by Life

Technologies). mRNA levels of HVEM, Gal-9, PD-L1 and IDO were normalized to the geomean of GUSB, PMM1 and HPRT1 mRNA levels. Primers for GUSB, PMM1, HVEM and PD-L1 were designed using the NCBI database. Primers for Gal-9 were obtained from PrimerBank (https://pga.mgh.harvard.edu/primerbank/). Primers for HPRT1 (Kim S et. al.. BioTechniques 2003; 35:456-460) and IDO (Stephen GL et.al. Am J Reprod Immunol 2015; 73: 36–55) were obtained from the literature. All primers for the mentioned targets are intron spanning (sequences shown in Supplementary Table 2).

Supplementary tables

Supplementary table 1. Primary antibodies used				
**Antigens**	**Antibody source**	**Clone**	**Retrieval buffer**	**Dilution**
PD-L1	Dr. G. Freeman[a]	405.9A11	Tris EDTA	1:50
Gal-9	R&D systems[b]	Goat polyclonal	Tris EDTA	1:200
HVEM	Millipore[c]	2G6-2C7	Citric acid	1:200
IDO	Millipore[d]	10.1	Citric acid	1:200
CD-8[e]	Dako[f]	C8/144b		

[a] Kindly provided by Dr. Gordon J Freeman, Dana-Farber Cancer Institute, Boston, MA
Choueiri TK et.al., Ann Oncol. 2014: 25:2178-84

[b] http://www.rndsystems.com/Products/AF2045
Mengshol JA et. al. PLoS One. 2010: 5:e9504

[c] http://www.merckmillipore.com/NL/en/product/Anti-TNFRSF14-Mouse-mAb-%282G6-2C7%29,EMD_BIO-AP1159?CategoryName=000000260002b67900020023&CategoryDomainName=Merck-MerckMillipore

[d] http://www.merckmillipore.com/NL/en/product/Anti-Indoleamine-2%2C3-dioxygenase-Antibody%2C-clone-10.1,MM_NF-MAB5412?bd=1#documentation
Soliman H et.al., Cancer Immunol Immunother. 2013: 62: 829–837

[e]CD8 staining performed under standard clinical laboratory conditions

[f]http://www.dako.com/nl/ar38/p102650/prod_products.htm?setCountry=true&purl=ar38/p102650/prod_products.htm?undefined&submit=Accept%20country
Mason DY, et.al., J Clin Pathol 1992:45:1084-8

Supplementary Table 2. Primers used			
Gene	Symbol	Forward primer	Reverse primer
Glucuronidase, beta	GUSB	5'-CAGGTGATGGAAGAAGTGG-3'	5'-GTTGCTCACAAAGGTCACAG-3'
Phosphomannomutase 1	PMM1	5'-CGAGTTCTCCGAACTGGAC-3'	5'-CTGTTTTCAGGGCTTCCAC-3'
Hypoxanthine phosphoribosyltransferase 1	HPRT1	5'-GCTATAAATTCTTTGCTGACCTGCTG-3'	5'-AATTACTTTTATGTCCCCTGTTGACTGG-3'
TNF receptor superfamily, member 14	HVEM	5'-CACCGAGAGTCAGGACAC-3'	5'-GAAACCACCATACCCAGTG-3'
Lectin, galactoside-binding, soluble, 9	Gal-9	5'-TCTGGGACTATTCAAGGAGGTC-3'	5'-CCATCTTCAAACCGAGGGTTG-3'
CD274	PD-L1	5'-GTGACCAGCACACTGAGAAT-3'	5'-CCAGAATTACCAAGTGAGTCC-3'
Indoleamine 2,3-dioxygenase 1	IDO1	5'-GCCAGCTTCGAGAAAGAGTTG-3'	5'-ATCCCAGAACTAGACGTGCAA-3'

Supplementary Table 3. Etiology of liver disease*	
Type of liver disease	N (%)
No known liver disease	50 (32.5)
Hepatitis B	30 (19.5)
Hepatitis C	30 (19.5)
Alcoholic liver cirrhosis	15 (9.7)
NASH	14 (9.1)
Hemochromatosis	5 (3.2)
Cryptogenic cirrhosis	3 (1.9)
Primary biliary cirrhosis	2 (1.3)
Porphyria	1 (0.6)

*When two etiologic factors where present in a single patient only the most dominant etiologic factor was considered, as determined by an experienced hepatologist. Thus the liver disease of seven patients with Hepatitis-B sero-positivity was attributed to other concurrent etiologic factors (Hepatitis-C x4, alcoholic liver cirrhosis x2, NASH x1). Etiologic information was missing from 4 patients.

Supplementary Table 4. Causes of death

Cause of death	N (%)
HCC	42 (27.3)
Postoperative complications (<3 months post HCC surgery)	14 (9.1)
Other cancer (not HCC)[1]	4 (2.6)
Other cause (not HCC)[2]	5 (3.2)
Alive	89 (57.8)

[1]Lung cancer x1, urothelial carcinoma x1, cholangiocarcinoma x1, colorectal cancer x1
[2]Severe lung disease x1, cardiovascular x2, pneumonia x1, unknown (sudden death) but not HCC (negative workup just before death) x1

Supplementary Table 5. Correlations of immune biomarker expression with clinicopathologic characteristics								
Clinicopathologic characteristics	PD-L1 Tumor	Gal-9 Tumor	HVEM Tumor	IDO Tumor	PD-L1 TFL	Gal-9 TFL	HVEM TFL	IDO TFL
Hepatitis-B	.247	.259	.540	.364	.459	.112	.210	.573
Hepatitis-C	.513	.334	.536	.411	.369	.288	.422	.056
Cirrhosis	.113	.571	.135	.045	.465	.032	.401	.024
Tumor differentiation	.429	.804	.768	.247	.963	.802	.541	.906
Vascular invasion	.122	.286	.408	.349	.008	.157	.174	.337
One vs multiple lesions	.446	.316	.045	.249	.246	.118	.509	.498
Tumor Size > 3cm	.315	.318	.037	.442	.413	.032	.109	.578
AFP>100 ug/l	.159	.157	.064	.065	.403	.297	.503	.105

Results provided as p-values. Note that after Bonferroni correction none of the associations reach statistical significance.

Supplementary table 6: Co-expression patterns of immune inhibitory molecules and CD8+ TIL counts in tumors. Tables 5a, 5b, 5c are 2x2 tables showing co-expression patterns of inhibitory molecules

Table 5d is a 2x2 table of CD8+ TIL count and PD-L1 expression

5a		Gal-9		p =.001
		Low	High	Total
PDL1	Low	**12**	**13**	25
	High	**20**	**101**	121
	Total	32	114	146

5b		HVEM		p <.001
		Low	High	Total
PDL1	Low	**8**	**16**	24
	High	**5**	**116**	121
	Total	13	132	145

5c		HVEM		p <.001
		Low	High	Total
Gal-9	Low	**10**	**22**	32
	High	**3**	**114**	117
	Total	13	136	149

5d		PD-L1		p =.046
		Low	High	Total
CD8	Low	**21**	**83**	104
	High	**3**	**38**	41
	Total	24	121	145

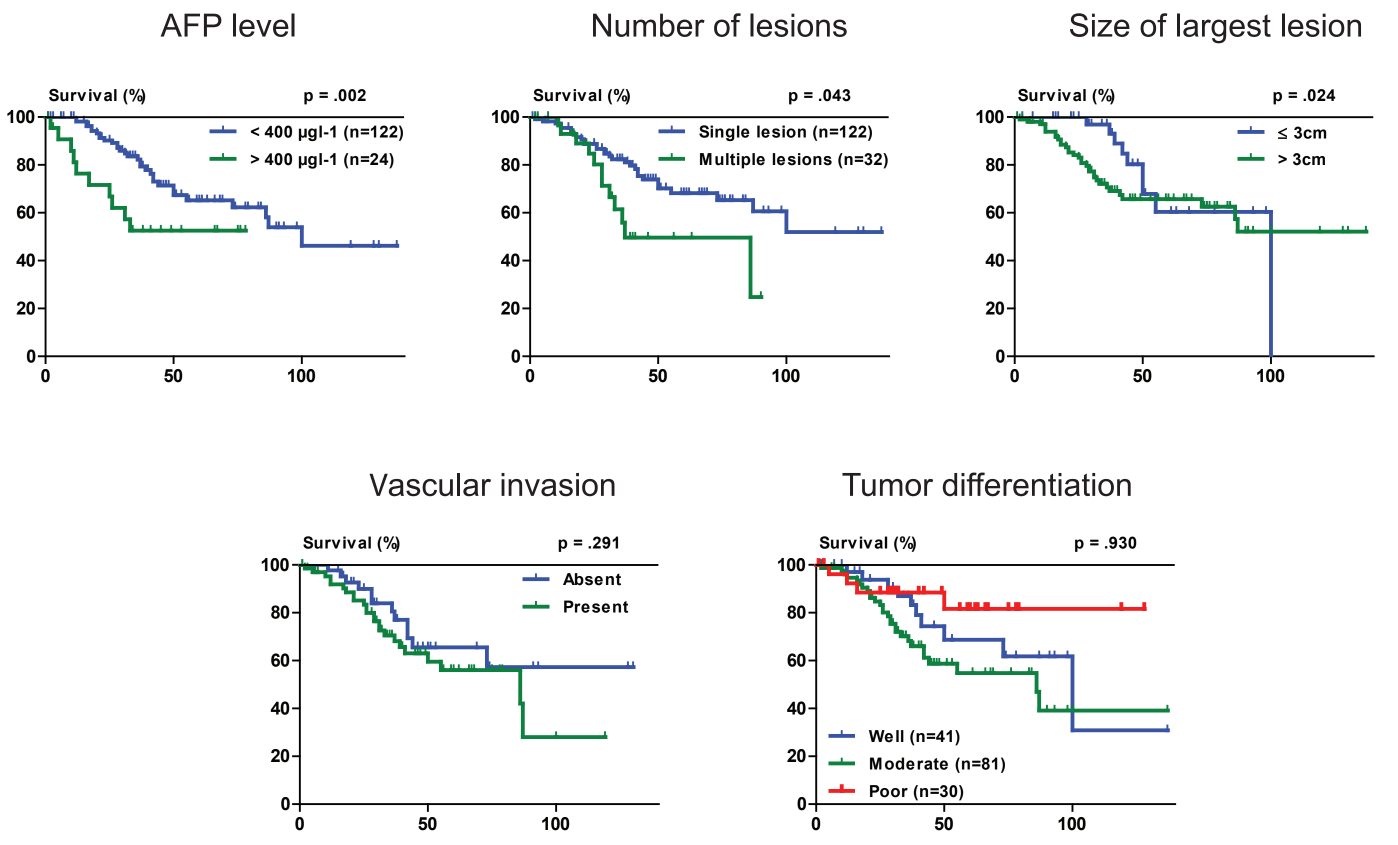

Supplementary Fig. 1. Kaplan-Meier curves of HCC-specific survival in relation to baseline clinicopathologic characteristics for the combined cohort of patients.

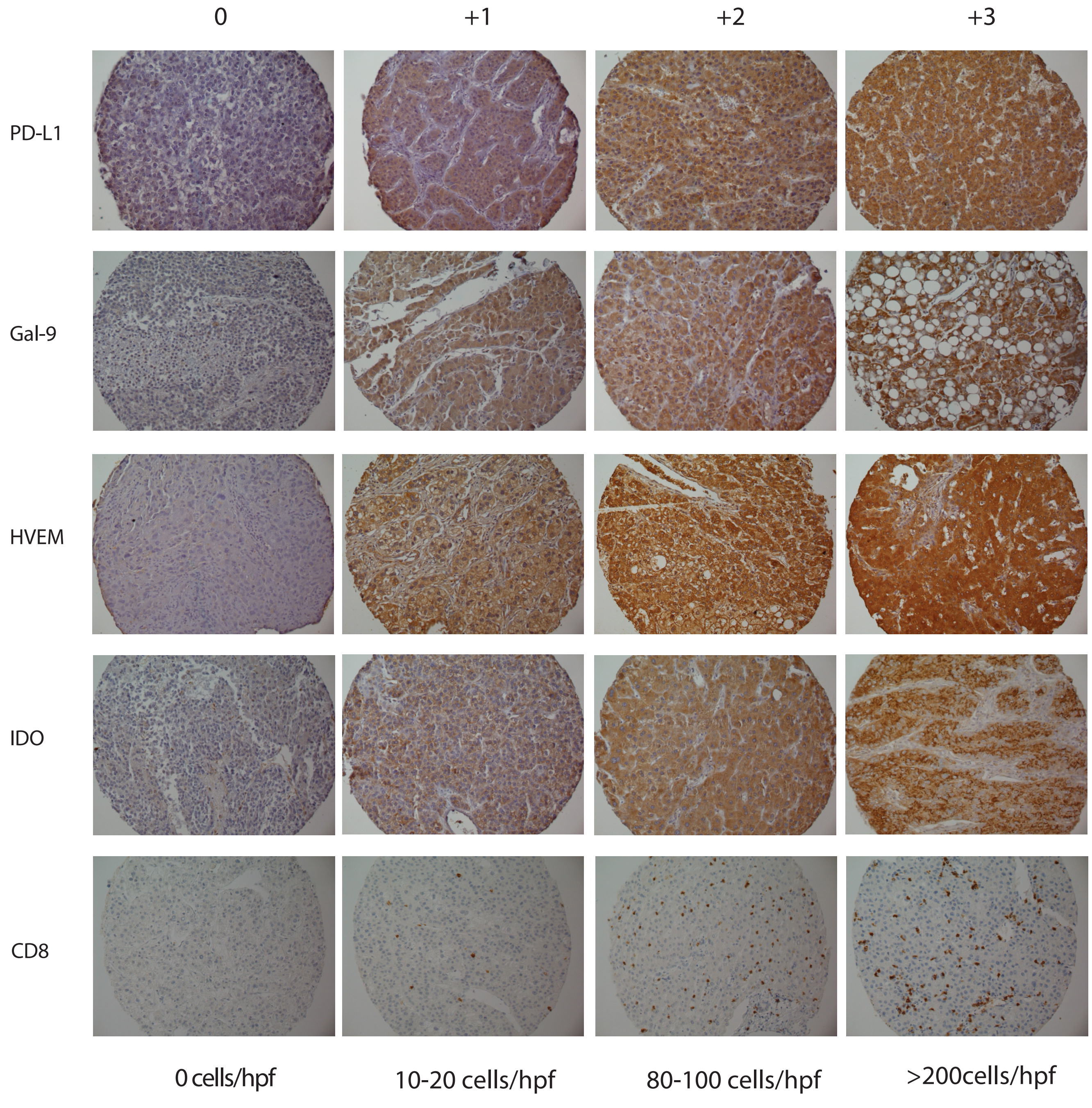

Supplementary Fig. 2. Representative immnochistomemical stainings, showing variable expression of immune inhibitory molecules and CD8+TIL infiltration, in tumor tissues.
Numbers in the top indicate intensity scores while numbers in the bottom indicate CD8+ counts per tissue core.

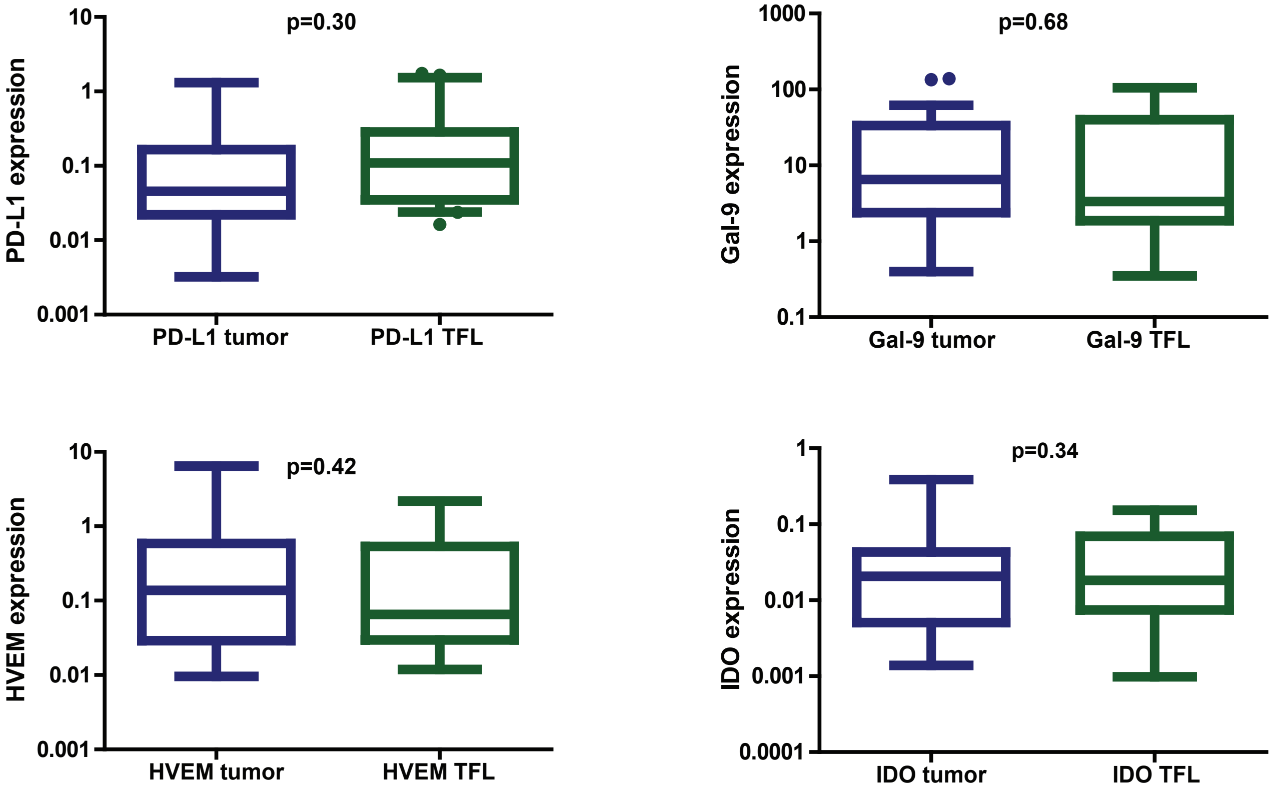

Supplementary Fig. 3. mRNA expression of PD-L1, Gal-9, HVEM and IDO in tumor and TFL tissue. Boxplot of mRNA expression levels in 20 HCC patients with available fresh frozen tissue from the tumor and TFL area. Real Time RT-PCR data are corrected with the geomean of three housekeeping genes: GUS, PMM1, HPRT1. Note that these 20 patients are not part of the study cohort.

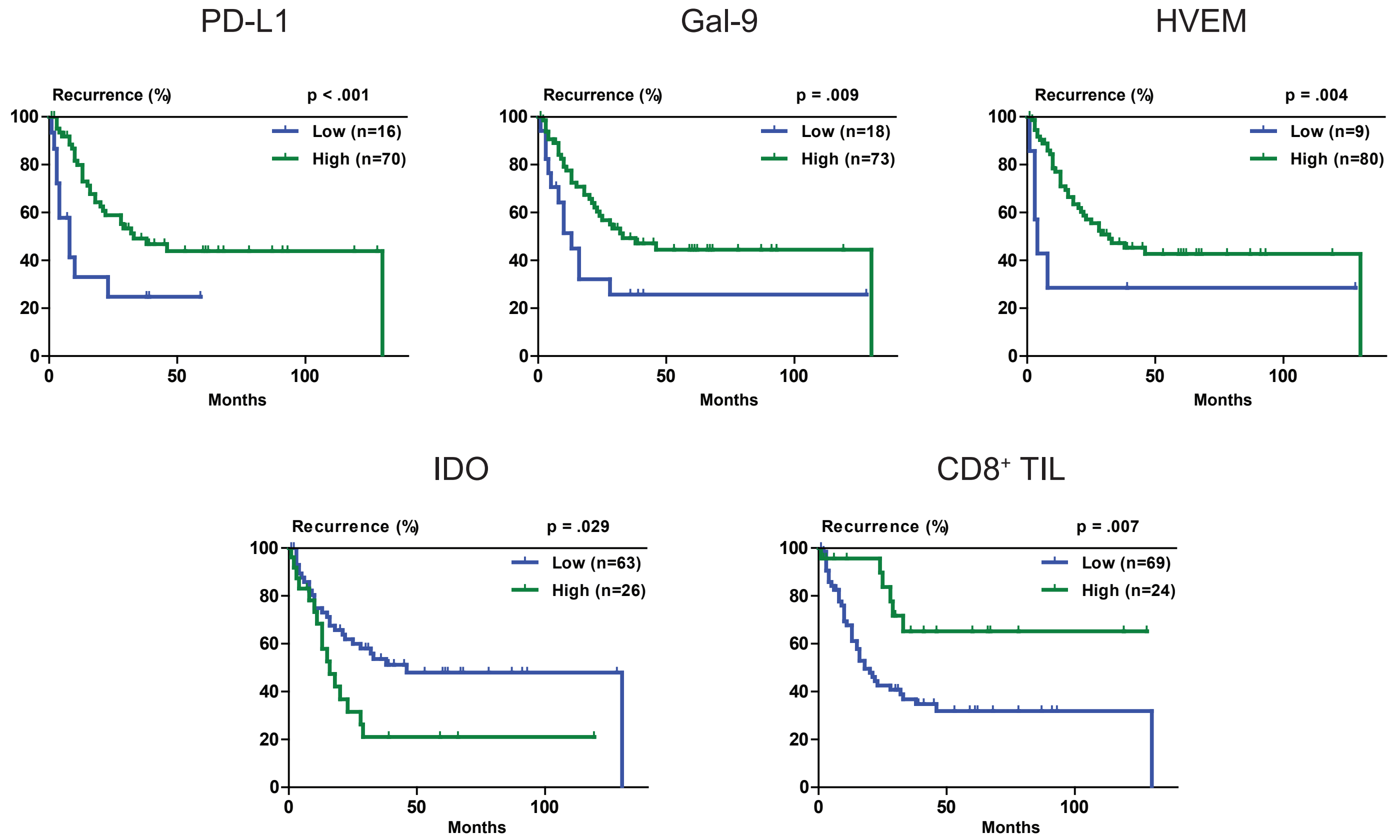

Supplementary Fig. 4. Kaplan-Meier curves of time to recurrence in relation to immune inhibitory molecule expression in tumors for the EMC cohort.

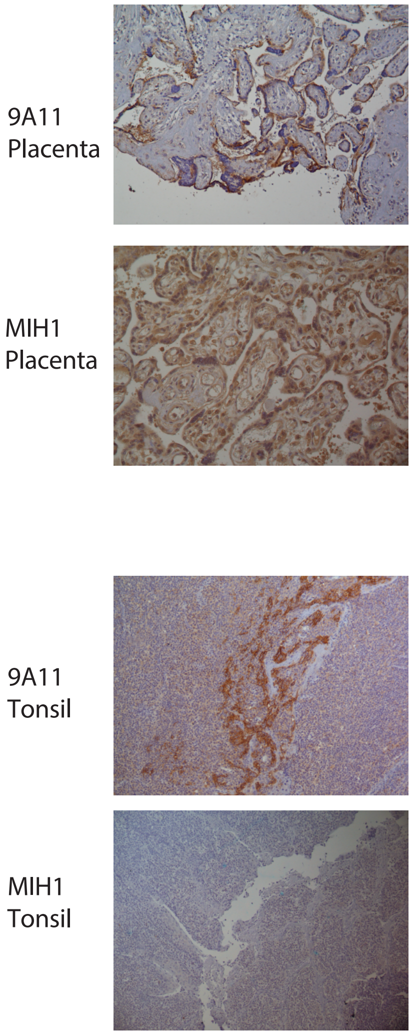

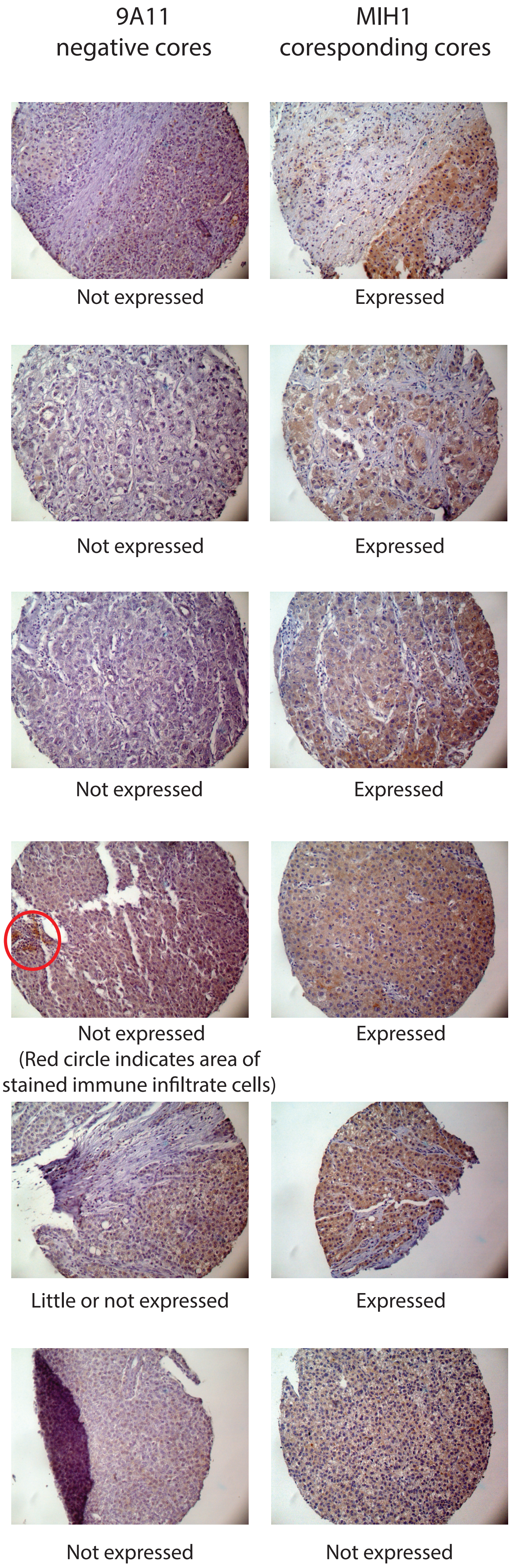

Supplementary Fig. 5. Comparison between the anti-PD-L1 antibody clones 9A11 and MIH1.

Note that the MIH1 antibody does not selectively stain trophoblastic cells in human placenta FFPE tissue or the crypt regions of human tonsil FFPE tissue. In addition, in 5/6 cores, HCC tumor cells not stained with the 9A11 clone are stained with the MIH1 clone. This indicates a lack of spesificity for the MIH1 clone.
